# mHealth: A Strategic Field without a Solid Scientific Soul. A Systematic Review of Pain-Related Apps

**DOI:** 10.1371/journal.pone.0101312

**Published:** 2014-07-07

**Authors:** Rocío de la Vega, Jordi Miró

**Affiliations:** Unit for the Study and Treatment of Pain - ALGOS, Research Center for Behavior Assessment, Department of Psychology and Institut d’Investigació Sanitària Pere Virgili, Universitat Rovira i Virgili, Tarragona, Spain; California Pacific Medicial Center Research Institute, United States of America

## Abstract

**Background:**

Mobile health (mHealth) has undergone exponential growth in recent years. Patients and healthcare professionals are increasingly using health-related applications, at the same time as concerns about ethical issues, bias, conflicts of interest and privacy are emerging. The general aim of this paper is to provide an overview of the current state of development of mHealth.

**Methods and Findings:**

To exemplify the issues, we made a systematic review of the pain-related apps available in scientific databases (Medline, Web of Science, Gale, Psycinfo, etc.) and the main application shops (App Store, Blackberry App World, Google Play, Nokia Store and Windows Phone Store). Only applications (designed for both patients and clinicians) focused on pain education, assessment and treatment were included. Of the 47 papers published on 34 apps in scientific databases, none were available in the app shops. A total of 283 pain-related apps were found in the five shops searched, but no articles have been published on these apps. The main limitation of this review is that we did not look at all stores in all countries.

**Conclusions:**

There is a huge gap between the scientific and commercial faces of mHealth. Specific efforts are needed to facilitate knowledge translation and regulate commercial health-related apps.

## Introduction

Healthcare systems worldwide are becoming exhausted; many demands are placed on them but resources are scarce. Healthcare costs are escalating and our public health systems seem to be incapable of satisfying the needs of a fast growing population [1]. In this scenario, what is known as mobile health technology or “mHealth” – that is, healthcare supported by mobile communication technologies – has undergone exponential growth in the last few years.

Mobile health technology can make healthcare more accessible and affordable for all. It has proven to be a good way of delivering high-quality healthcare services to a variety of patient populations, particularly those with low incomes [2] and in remote places (far from reference centers) [3]. mHealth technology has also proven to be highly suitable for young people (and also very popular) [4] as they spend more time using electronic media than doing any other activity besides sleeping [5].

It has been estimated that by the end of 2016, there will be ten billion mobile devices in use around the world [3]. Patients and healthcare professionals are increasingly using health-related applications [6]. To date, more than 97,000 of these applications have been developed and in the next few years more than three million free and 300,000 paid downloads are expected to be made of mHealth applications just in the USA [7]. A recent study concluded that the Smartphone is the most popular technology among physicians since the stethoscope [1]. Furthermore, mobile phone use seems to be greater among those populations most in need of such interventions [8]. mHealth seems to be a logical, acceptable, and affordable way to extend and improve health care.

Although the progress of mHealth has many advantages, some of which have been summarized above, this extremely fast growth also has a negative side: namely, most of the procedures available have not been subject to a thorough assessment and validation [9], [10]. Explicit and sensible concerns about ethical issues, bias, conflicts of interest [11], and security and privacy problems [2] have been raised in the specialized literature.

Some action protocols and strategies are being developed to deal with these as yet unsolved issues in Europe [12], [13] and the USA [14], [15]. For example, the World Health Organization in partnership with the United Nations specialized agency for information and communication technologies has developed an initiative regarding the management of Non-Communicable Diseases using mHealth [16]. Also, some charities, and not-for-profit or private organizations have launched initiatives to boost the potentialities of mHealth. This is the case, for example, of the mHealth Alliance, hosted by the United Nations Foundation [17]. Similarly, PatientView has recently released the web page “myhealthapps.net”, recommended by the Directorate General for Communications Networks, Content and Technology of the European Commission. This web page is an evolution of the previously published “European Directory of Health Apps” [18], in which patients’ associations from all over the world used a zero-to-five Likert-type scale to rate 307 health-related apps on the extent to which they help control their condition, keep them healthy, are trustworthy, are easy to use, allow them to network with people like them/who understand them, and can be used regularly. In the context in which we find ourselves, then, commercial apps are developing exponentially, while mHealth-related scientific publications are also growing. However, it is not clear that both worlds interact and, if they do, how. That is to say, is the growth rampant, or is there fruitful interaction between the two worlds? Are research findings translated and used to improve the apps that are created or are knowledge transfer processes failing?

In this situation, it would be extremely useful if a review were to map out the terrain, identify problems and tentatively suggest avenues for improvement.

However, the field of mHealth is so wide that a complete review and analysis cannot be contemplated. Therefore, we decided to focus on pain-related apps as a way of managing an otherwise insurmountable amount of information. First, although mHealth uses various alternatives and technologies to educate patients, and to prevent and/or treat illness, apps are at the heart of the process. Two specific features of apps make it particularly important for their quality and scientific rigor to be studied: namely, (1) the app is available to consumers who do not have a professional to recommend, prescribe or even monitor how they use it, and (2) too often there is nobody “responsible” and available if the app is not working as expected or if something goes wrong. Second, we decided to concentrate on pain-related apps because pain is one of the most generalized symptoms of chronic health conditions [19]. It is a ubiquitous health problem, and well suited to be assessed and managed with these mHealth interventions [20]–[22]. So it can be readily used to explore and exemplify the issues when looking into the current state of development of mHealth.

The general aim of this paper is to provide an overview of the current state of development of mHealth. In order to do so, and to exemplify the issues, we conducted a systematic review of the pain-related apps available and reported on their characteristics; we looked both at the commercial and the scientific aspects of this development. The specific objectives of our review are to: (1) detect the number of pain-related apps reported in scientific databases, (2) find out which ones are available at the stores for general consumers, (3) identify which pain-related apps are available at the main apps shops, (4) find out which of these apps are scientifically supported, and (5) uncover any other additional support that the apps may have.

Our specific hypotheses were that (1) only a few of the apps reported in peer-reviewed publications are available to the consumer, and (2) of the apps available in the shops, very few have a scientific base.

## Methods

### Phase I: what can be found in scientific databases?

#### Search strategy and selection criteria

Preferred Reporting Items for Systematic Reviews and Meta-Analyses (PRISMA) guidelines [23] were followed. Data for this review were identified by searches of following scientific databases: Medline (National Library of Medicine), Science Citation Index Expanded (Web of Science), Health Reference Center Academic (Gale), Wiley Online Library, American Psychological Association (Psycinfo), SciVerse ScienceDirect (Elsevier), SpringerLink, Wolters Kluwer - Ovid - Lippincott Williams & Wilkins (CrossRef), Directory of Open Access Journals (DOAJ), Social Sciences Citation Index (Web of Science), Taylor & Francis Online - Journals, Expert Reviews (Future Science), Informa - Informa Healthcare (CrossRef), SpringerLink Open Access, Wolters Kluwer - Ovid (CrossRef), BMJ Journals, DiVA - Academic Archive Online, Informa (CrossRef), and references from relevant articles using the search terms (Pain OR *ache) AND (Smartphone OR app OR application OR electronic OR “Personal Digital Assistant” OR PDA). Only peer-reviewed articles published in English or Spanish between 1996 (the release date of the first palmtop computer [24]) and December 2013 were included.

### Phase II: what scientifically assessed pain-related apps are available in the stores?

The name of each app retrieved in phase I was searched for in each of the following shops: App Store (iPhone), Blackberry App World, Google Play (Android), Nokia Store and Windows Phone Store.

### Phase III: what can be found in the stores?

In December 2013, the main Smartphone application shops were reviewed: App Store (iPhone), Blackberry App World, Google Play (Android), Nokia Store and Windows Phone Store. The review was conducted in the following countries: Canada, Spain, and USA. The search terms were: “Pain”, “*ache” and “dolor”. The applications (designed for both patients and clinicians) focused on pain education, assessment and treatment were included.

### Phase IV: what support do the apps available in stores have?

A step-by-step sequential strategy was followed to assess the quality of the apps found in phase III. First, the name of each app was searched for in the same databases as in Phase I. Then, the web page “myhealthapps.net” was also reviewed. All the pain-related apps were recorded. Finally, the name of each app was Google searched for such information as whether the developers had a webpage, which research centers used the app, who its creators were and/or the results it had provided, etc. This information was compared with the information obtained in phase I to see if the authors of the apps were the same as the authors of the publications.

## Results

### Phase I: what can be found in scientific databases?

**Figure 1:**
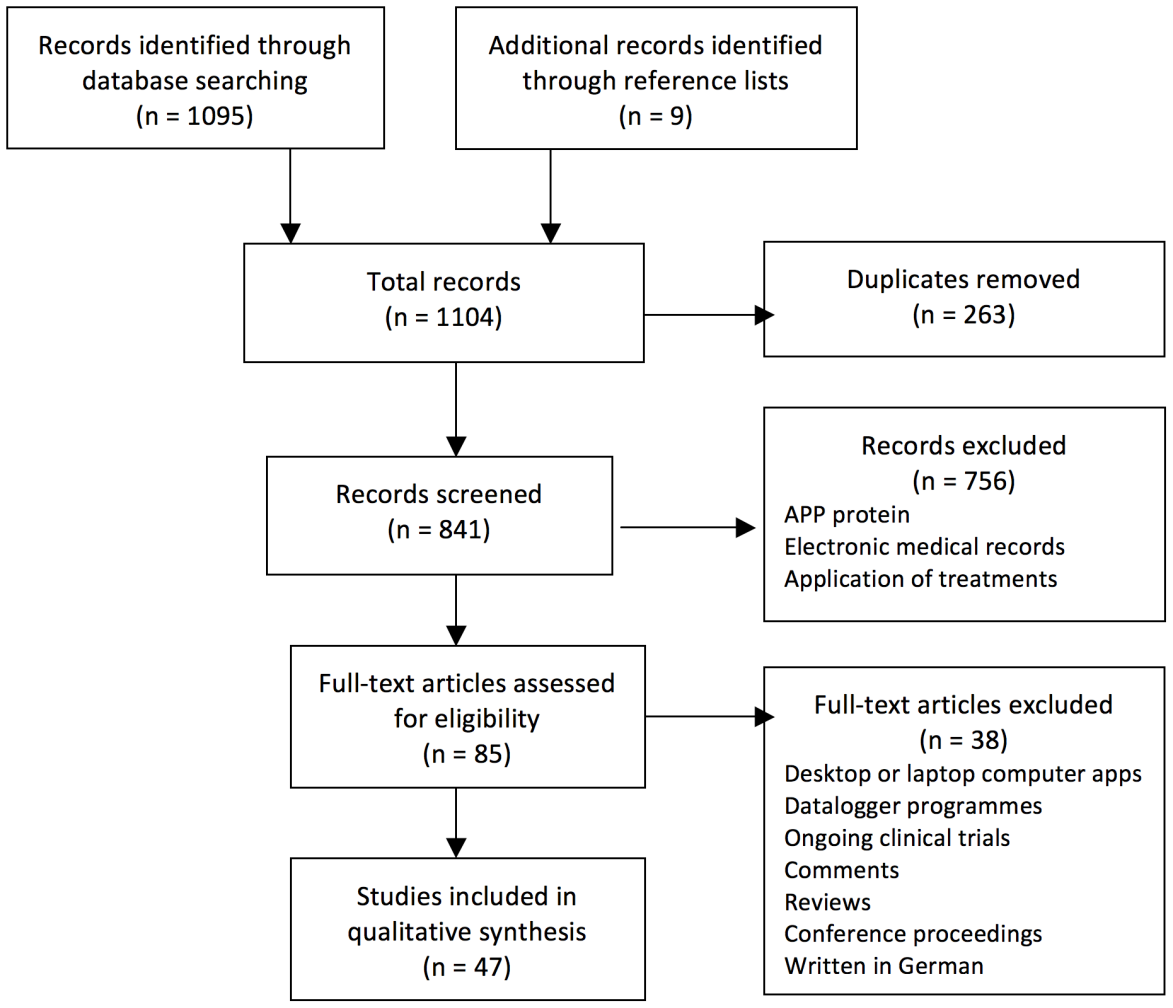
Flow chart of our systematic review selection process.

After reviewing the databases, we found 47 papers reporting on 34 pain-related apps. Figure 1 describes our study’s selection process.

As can be seen in Table 1, all apps are related to assessment, and almost all are available in English (26, 76.5%) and address non-specific chronic pain problems (28, 82.4%). About two-thirds are designed for adults (22, 64.7%).

**Table 1:** Apps reported on the scientific databases.

Author/s and year	App name	Domains	Pain problem	Targeted population	Language/s	App properties	Device
Aaron et al., 2005, 2006 [25], [26]Turner et al., 2005 [27]	Not reported	Pain intensity, pain-related activity interference, jaw use limitation and pain-coping strategies (cognitive coping, relaxation, activity reduction, and emotional support).	Chronic temporo-mandibular pain	Adults	English	Reliability (Cronbach’s α range, 0.64 to 0.88) and validity were demonstrated for all the scales.	PDA
Affleck et al., 1996 [28]	Not reported	Sleep quality, pain intensity, and attention to pain.	Fibromyalgia	Adults	English	Not reported	PDA
Allena et al, 2012 [29]	Not reported	Sleep time, presence of aura symptoms, Pain: time onset, intensity, side, type, presence of associated symptoms, medication use, and trigger factors.	Headaches	Adults	The study was conducted in Italy but a figure shows an English written diary	Easy to understand and to use. 98% completion rate.	PDA
Alfvén et al; 2010 [30]	SMS-pain-diary	Pain intensity, pain duration, and functional impairment.	Chronic pain	Children and adolescents (9–15 years old)	Norwegian	High construct validity (concordance of 0.77)High test-retest reliability (K = 0.73)Compliance: 75–83%Acceptability: easy to understand and use.	SMS-delivered diary
Chen et al., 2004 [31]	Not reported	PDA-based data management system	Acute Pain	Staff of an Acute Pain Service	English	User satisfaction, ease of access to drug reference and clinical guidelines were similar between the PDA and paper systems.	PDA
Connelly et al., 2010 [32]	Not reported	Headaches: occurrence, duration, and intensity.Child negative affect (PANAS-C)Weather variables	Headaches	Children and adolescents (8–17 years old)	English	80% completion rate	PDA
Connelly et al., 2010^a^, 2012 [33], [34]	Not reported	Assessment of: pain characteristics, activity limitations (Activity Scale for Kids), intensity of positive and negative emotions (PANAS-C) and emotion management (Children’s Emotion Management Scale).	Juvenile Idiopathic Arthritis	Adolescents (8–18 years old)	English	Rates of compliance: 41% to 100%Electronic version of the “Activity Scale for Kids” showed strong internal consistency (Cronbach’s α = 0.88–0.94)	Smartphone’s screen optimized e-diary, not properly an app itself.
Evans et al., 2007 [35]	Not reported	Pain data: Gracely pain scale, study medication dosing, rescue medication use and sleep quality.	HIV-associated sensory neuropathies (HIV-SN)	Adults	English	90% completion rate.	PDA
Gaertner et al., 2004 [36]	Not reported	MIDOS for pain and symptom assessment.	Cancer and non-cancer chronic pain	Adults	English	No significant difference with paper diary on pain and symptom intensity.It was used more frequently.Good patient satisfaction.	PDA
Ghinea et al., 2008 [37]	Not reported	Pain location, type (numbness, pain, pins and needles, and ache) and intensity using a 3D mannequin, time of input.	Back pain	Adults	English	Good acceptability and usability results in clinicians and patients. Finer division of the body mannequin suggested.	PDA
Goldberg et al., 2007 [38]	Not reported	Presence of headache symptoms, pain intensity, localization and quality, related symptoms, interference and premenstrual symptoms.	Menstrually related headache	Adult females	English	Difficulties with the PDA were encountered.35% of abnormal session endings.	PDA
Goldstein et al., 2003 [39]	Not reported	Postoperative pain measured by the number of pills taken and patient return to work.	Hernia	Adults	English	Not reported	PDA
Gulur et al., 2009 [40]	CFS	Pain intensity and mood state.	Acute pain	Children and adolescents (3–17 years old)	English	Good feasibility: children were able to use the CFSAdequate test-retest reliability for both pain (r_1_ = 0.77, r_2_ = 0.80) and mood (r_2_ = 0.82).High concurrent validity (r_S_ = −0.68)Adequate discriminant validity (r = 0.55)77% of children preferred the CFS to the WBFS.	PDA
Heiberg et al., 2007 [41]	Not reported	VAS for pain, fatigue, and global disease; the Rheumatoid Arthritis Disease Activity Index; the Short Form 36 and Modified Health Assessment Questionnaire	Rheumatoid arthritis	Adults	Norwegian	The average scores and measures of variation did not differ significantly between PDA and paper diaries.The completion was similar.82.9% preferred using PDA.	PDA
Jacob et al., 2012, [42] 2013 [43]	Not reported	Assessment of symptoms, pain intensity, medication, non-pharmachological strategies, sleep, feelings/thoughts, fluids and healthcare use.	Sickle cell disease	Children and adolescents (10–17 years old)	English	Allows accurate symptom assessment.It is easy to use and efficient to complete.	Smartphone’s screen optimized e-diary, not properly an app itself.
Jamison et al., 2002 [44]	Electronic VAS	Assessment of pain intensity (VAS).	Healthy volunteers	Adults	English	High correlations between electronic VAS and paper VAS scores for both cognitive (verbal intensity) and sensory (weight) stimuli (r = 0.91).	PDA
Jamison et al., 2001, [45] 2006 [46]	Not reported	Pain, mood, activity, medication, and side effects.	Chronic low-back pain	Adults	English	High degree of agreement between electronic diary and telephone-collected data.	PDA
Jibb et al., 2012 [47]Stinson et al., 2013 [48]	Pain Squad	Assessment of pain and cancer-related symptoms.	Cancer	Children and adolescents (8–18 years old)	English	Good usability and feasibility resultsHigh rates of compliance (81%)	iPhone
Johnson et al., 2010 [49]	EPTAD	Assessment of routine pain, acute pain episode, routine medication and non-medication treatment, sleep.	Non-cancer chronic pain	Adults	English	Poor usability results: screen and font size were found acceptable but navigation problems were found.	PDA
Junker et al., 2008 [50]	Electronic version of VAS and the pain DETECT questionnaire	Pain severity: average and worst over the last 2 weeks, present and symptoms of nociceptive pain (painDETECT).	Chronic pain	Adults	English	High correlations between electronic and paper measures.	PDA
Kristjánsdóttir et al., 2011, [51] 2013 [52], [53]	Not reported	Diaries and daily situational feedback.	Chronic widespread pain	Adults	Norwegian	Moderate improvements in catastrophizing and acceptance.Moderate rates of compliance (66.7%)	Smartphone’s screen optimized website, and some audio files included in a Smartphone.
Lewandowski et al., 2009 [54]	Not reported	Pain intensity, pain location, activity restriction, and depression.	Chronic pain	Children and adolescents (8–16 years old)	English	Greater compliance (98%) with the electronic format (mean of 6.89 days completed) in contrast to the paper format (mean of 4.97 days completed)	PDA
Marceau et al., 2010 [55]	Electronic version of BPI, PCS, ODI, CES-D	Assessment of pain history, intensity, location, interference with daily activities, and mood (BPI); rumination, magnification, and helplessness (PCS); disability (ODI); depression (CES-D).	Non-cancer chronic pain	Adults	English	All the patients were able to complete the diaries.Good acceptance rates by both patients and doctors.	PDA
McClellan et al., 2009 [56]	Daily Pain and Activity Diary	Pain location and severity, sleep quality, functional activities, use of medication, and coping skills.	Chronic pain	Children and adolescents (8–20 years old)	English	Usability and feasibility:High daily diary completion (no incomplete data, 100% of items completed)Both parents and children rated the diaries as easy to use.	PDA
Palermo et al., 2004 [57]	Not reported	Pain and distress ratings (occurrence, location, intensity, duration, and emotional upset), somatic symptoms, and activity limitations.	Headaches and Juvenile Idiopathic Arthritis	Children and adolescents (8–16 years old)	English	Greater compliance compared with a paper diary (83.3% vs. 46.7%)Greater accuracy compared with a paper diary (100% vs. 51.3%)No differences in acceptability depending on the diary format.	PDA
Peters et al., 2000 [58]	Not reported	MPI (pain severity, interference of pain, affective distress, social support as well as punishing, solicitous and distracting responses to the pain problem by the spouse), the SF-36 (physical functioning, role functioning, vitality) and CSQ (catastrophizing, denying/ignoring pain, positive self-talk and diverting attention), sleep quality, sickness leave, medication and satisfaction with role functioning.	Unexplained pain	Adults	Dutch	88% completion rate.MPI scales with equivalent diary items (range: r = 0.33–0.53).SF-36 and the diary correlated highly (r = 0.73).CSQ and the diary: catastrophizing (r = 0.66), diverting attention and ignoring/denying pain (r = 0.41).No evidence of instrument reactivity was found.	PDA
Roelofs et al., 2004, [59] 2006 [60]	Not reported	Current pain intensity, attention to pain, passive attention to pain, additional questions (not specified).	Chronic low-back pain	Adults	English	72.7% completion rate.	PDA
Sorbi et al., 2006 [61], [62]	Not reported	Pain intensity, fear-avoidance, cognitive and spousal solicitous, and punishing pain responses.	Chronic pain	Adults	Dutch	A pilot study in 4 patients: feasibility and patient acceptability.86–93% completion rate.	PDA
Sorbi et al., 2007 [63]Kleiboer et al., 2009 [64]	Not reported	Migraine headache, medication use, attack precursors, self-relaxation and other preventive behavior, menstruation, and disturbed sleep.	Migraine headache	Adult females	Dutch	Feasibility: minimal technical problems, good compliance, and successful execution.Acceptability: positive participant responses concerning usefulness, supportiveness, and low burden.	PDA
Stinson et al., 2006, [65] 2008 [66], [67]	e-Ouch electronic diary	Pain intensity, number of painful joints, number of word descriptors, pain unpleasantness, interference (e.g. activities, mood, sleep), stiffness and tiredness, control over pain.	Arthritis	Children and adolescents (8–18 years old)	English	Good usability, feasibility, validity and sensitivity to change properties.	PDA
Stone et al., 2003 [68]	Not reported	Pain: intensity (rated on a 100-point VAS), sensory characteristics, affective responses, and degree that activities were limited by pain. Additional questions about place, activity, and mood.	Chronic pain	Adults	English	94% completion rate.Little difficulty and burden with the diary was reported.	PDA
VanDenKerkhof et al., 2003 [69]	Not reported	Standard pain scoring systems vand an extensive list of drug-related side effects.	Acute Pain	Staff of an Acute Pain Management Service	English	PDA assessments were more likely to report pain and side effects. The median time of the assessment was 53 sec longer using the PDA but the median time of the full visit was 74 sec shorter.	PDA
Walker et al.; 2002 [70]	Not reported	Gastrointestinal symptoms: abdominal discomfort, bowel dysfunction, extent of discomfort, frequency of bowel movements, and stool consistency.	Gastrointestinal pain	Children (6–10 years old)	English	UsabilityEasy to learn, quick to use and understand. No interference with family activities. Children needed little assistance in answering the questions.FeasibilityAccuracy: Responses were “very accurate” or “accurate”.High level of satisfaction.Compliance: 100% (no missing data).	PDA
Wood et al., 2011 [71]	Electronic version of FPS-R	Assessment of pain intensity (FPS-R).	Postoperative disease-related pain	Children (4–12 years old)	Pictures	High agreement (K = 0.85) and high correlation (r = 0.91) between electronic and paper versions.No mean difference between the two versions (3.1±2.3 for paper and 3.2±2.3 for electronic version).The electronic version was preferred by 87.4% of the children.	PDA

a It is an earlier version, slightly different from the final version.

PDA: Personal Digital Assistant; SMS: Short message service; PANAS-C: Child version of the Positive and Negative Affective Schedule; MIDOS: minimal documentation system; CFS: Computer Faces Scale; WBFS: Won Baker Faces Scale. VAS: Visual Analogue Scale; EPTAD: Electronic pain treatment activity diary; BPI: Brief Pain Inventory; PCS: Pain Catastrophizing Scale, ODI: Oswestry Disability Index, CES-D: Center for Epidemiologic Studies-Depression Scale; MPI: Multidimensional Pain Inventory; CSQ: Coping Strategy Questionnaire; FPS-R: Faces Pain Scale – Revised.

### Phase II: are the scientifically assessed apps available in the stores?

No pain-related app reported in any paper found during Phase I was available in any of the five main shops for the general public.

### Phase III: what can be found in the stores?

A total of 283 pain-related apps were found in the five shops searched. Because of word count and space limitations, the full list is provided as an annex to the article (see Table S1).

### Phase IV: what type of support do the pain-related apps available in stores have?

**Figure 2:**
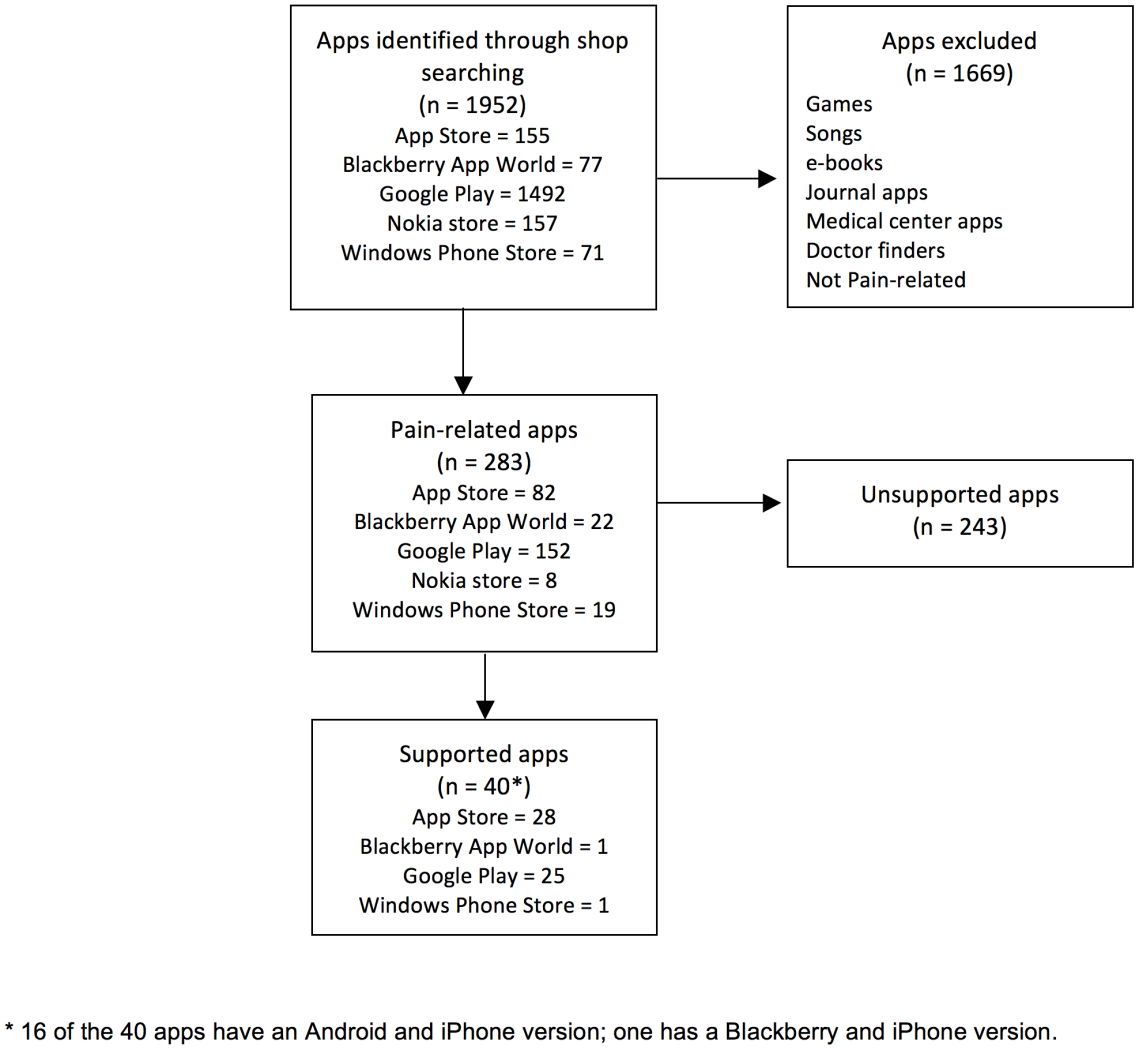
Flow chart of pain-related apps selection process.

When we searched for these 283 apps in the scientific databases, we did not find a single article that was related to them in any way. Therefore, this search found no evidence of scientific support for the 283 pain-related apps. Nevertheless, some apps do have other support types. Figure 2 describes our app selection process.

**Figure 3:**
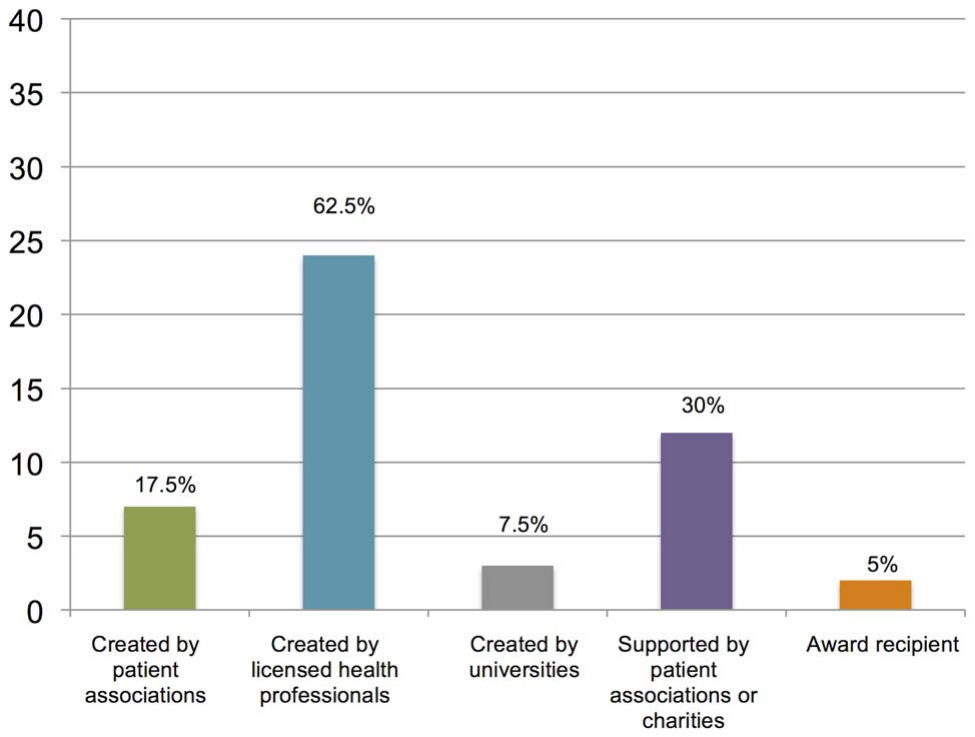
Type of support that the pain-related apps have.

A full description of 40 apps – including name, developers, supports, pain problem it addresses, features, platform, price, language/s and user ratings – is provided as an annex to the article (see Table S2). Figure 3 summarizes the type of support that the pain-related apps have.

Most of the apps are available in English (36, 90%), and have been developed in the USA (16, 40%), the EU (15, 37.5%), or Canada (6, 15%). The App Store and Google Play are the most important platforms, hosting 39 (97.5%) of the supported apps. The most important sources of support to these apps are: having a licensed professional as a creator (24, 62.5%) or being recommended by a patient association (12, 30%). “Pain in general” (9, 22·5%), followed by back pain (8, 20%), headache (7, 17.5%) and arthritis (6, 15%), are the types of pain that these apps are most commonly designed for. As far as the targeted consumers are concerned, most of the apps are addressed to patients (28, 70%) and only a few have been developed for healthcare professionals (5, 12.5%) or both audiences (7, 17.5%). Most patient-oriented apps provide information about the pain problem/illness and ways to check symptoms and track medication consumption. Only a few provide information about alternative ways of coping with the health problem either through videos or written instructions, for example, about exercising, massage, or even hypnosis. Professional-oriented apps provide support for diagnosis, medication dose calculation, or self-report questionnaires. All patient-oriented applications are classified as +4 years or “low maturity”, while professional-oriented are classified as +17 years.

None of the authors/developers of the apps were found to be the authors of articles about them.

## Discussion

Overall, this review indicates that the commercial and scientific sides of the mHealth coin do not interact properly. We found that pain-related apps that have been reported in scientific journals have not yet made their way into the shops and are therefore unavailable to clinicians and/or patients. Conversely, 283 pain-related apps were available in the main shops, but none of them had been scientifically validated or proven to be effective. These findings are in line with our hypotheses but the situation is even more extreme than we had imagined. However, it may be just a matter of time before this state of affairs changes because some apps are currently in the last stages of the knowledge translation process. For example, *Painometer V2*, an app developed to help with the assessment of pain intensity is already available in Google Play and has shown some evidence of usability [72], [73] and of the psychometric properties of the scales contained [74]. Pain Squad is another app that has already reported information on usability, feasibility, and compliance [47], [48]. It is currently available in four Canadian hospitals and may be available soon at the App Store [75].

mHealth technologies have numerous important advantages over other more traditional alternatives. For example, they capture time- and date-stamped information, and provide detailed and non-biased information on such fundamental health-related variables as physical activity or physiological responses, thus reducing memory bias. They can also be extremely useful in public health actions (for example, by providing routes to help patients who have to take medications on a specific schedule) and help us reach underserved populations, those that are most in need of health care support.

In the midst of this *huge*, positive development there are some fundamental concerns that require appropriate responses. For example, issues of confidentiality or the protection of patients’ personal data still have to be dealt with. Furthermore, some apps occupy a “legal void”. For example, electronic diaries or cognitive-behavioral treatments for health conditions are unregulated, a situation that needs to be remedied. Overall, the results of this review indicate that consumers run some risks above and beyond paying for a potentially useless app. For example, we found some apps that claimed they could heal the body by emitting vibrations, “brain waves”, or accessing the subconscious to “tell the body to heal”. These unproven claims may lead patients to a feeling of helplessness and lack of control about their illnesses.

As mentioned above, there is a gap between the scientific and the commercial sides of the mHealth coin. Significant developments have been made in both areas but they remain essentially disconnected, advancing in parallel with no significant interaction. None of the apps in the shops have proved to have scientific support and only a fifth (57, 40+17 versions for other platforms, 20.1%) of them have some type of support. Some scientifically developed apps look promising but there is an urgent need to promote actions for knowledge translation in this field. Other researchers have found similar results when looking into other mHealth areas: apps to manage diabetes [76] and the world deadliest diseases [77]. They both found that the commercial area was significantly more developed than the research field. Referring to cardiology apps [78], they found that most of the published papers reviewed monitoring apps, but similarly to our findings, the majority was not smartphone apps themselves but computers apps that could be also used by a mobile phone or a smartphone.

In the near future, perhaps, physicians will be prescribing specific applications to specific patients for specific problems [79] (very much like today when they electronically prescribe medications, or work with the patient’s electronic clinical history system and health records). It does not make much sense for drugs to have to go through a long and complex process between the discovery of the active ingredient and being put on the market, while apps do not have to fulfill any requirements at all, not even show that they are effective and safe. There may be no need for health-related apps to go to the extremes of approved drugs, but a minimum level of quality should be compulsory. Health-related apps can also have negative effects. Therefore, we should be able to regulate what is available in stores, and prevent unregulated apps from being published in the field of health (health-related apps should inform about quality controls and prove they are efficacious before they can use the adjective health, in the same way that current laws prevent food from bearing the name “bio” if their real properties have not been subject to strict analysis). Furthermore, lists of approved health-related apps ought to be published and the general public informed, for example, through an app-related vade-mecum, so that both health experts and patients can make informed decisions about whether to use certain apps. A promising avenue that would prove fruitful in the near future is the work done by Public Agencies in the field of quality distinctions, for example, the “AppSaludable Distinctive”, reported in the last European Journal of e-practice [80] To date, and to the best of our knowledge, no pain-related app has been awarded this quality stamp and just one (*Painometer v2*) has applied for it [81].

Perhaps the most important limitation of this review is that we did not look at all stores in all countries. We selected three of the possibilities, not only because it was convenient, but also because it was what could be feasibly done. Our hypothesis is that if we had conducted specific reviews for the 97,000 health-related apps available worldwide, results would not have been much different, particularly considering that we explored the most important app stores and that other researchers [76]–[78] found similar results.

All the articles reviewed were related to pain assessment, with some dealing with educational issues. Future studies are needed in the area of pain management. We are aware that some research groups are working on this subject, so we can expect developments in the future. Most apps are designed for adults or adolescents, but there are very few for children. However, children are using these technologies at a very early age: 72% of children younger than eight years old use mobile devices and 50% of those use apps [82]. Therefore, additional research is greatly needed in this area if health-related apps are to be developed that are efficacious and developmentally appropriate.

## Supporting Information

Table S1 Pain apps available in the main five shops.
(DOCX)

Table S2 Characteristics of the commercial apps that have some sort of support.
(DOCX)

Protocol S1 Protocol for the systematic review.
(DOCX)

Checklist S1 PRISMA Checklist.
(DOC)
