# Supplementary material for: mHealth: A Strategic Field without a Solid Scientific Soul. A Systematic Review of Pain-Related Apps
Source: PLoS One. 2014 Jul 7;9(7):e101312. doi: 10.1371/journal.pone.0101312 (PMC4085095; doi:10.1371/journal.pone.0101312)
Supplement: Table S1 — Pain apps available in the main five shops. (DOCX) [file pone.0101312.s001.docx]

**Table S1. Pain apps available in the main five shops**

**S1.A App store (iPhone and iPod)**

| **App Name** | **Language** | **Developer** |
| --- | --- | --- |
| Ache relief | English | S Liu |
| Ache Break | English | Lita Van Wagenen |
| Acupressure: Treat Yourself | English, Spanish | Alexander Mokrushin |
| Afa-MICI | Czech, Dutch, English, French, German, Italian, Japanese, Korean, Polish, Portuguese, Russian, Simplified Chinese, Spanish, Swedish, Traditional Chinese, Turkish | De YLLY |
| AGMethod | English | Cristiano Campanella |
| Alivia las contracturas | Spanish | Sebastian Chab |
| Analgesia Pràctica | Spanish | Juan Pablo Burgu  Urologia practica CB |
| Aps SmartCal | Spanish | MEED COMUNICACIÓN SL |
| ArthritisID | English, French | ACE Planning and Consulting Inc, Canada |
| Aviación Extensiones | Spanish | DawnSun Technologies LLC |
| Back Pain | English | Ortho Tech Inc |
| Bebesincolico | Spanish | El Puericultor SLNE |
| Chronic Pain Tracker | English | Chronic Stimulation LLC |
| CountH | English | Sadao Kobayashi |
| Dario del dolor- Catch my pain | English, Spanish | Sanovation AG  Zurich University |
| Dental Emergency | English | Paul Speziale |
| Diagnosaurus Ddx | English, French, simplified Chinese | Unbound Medicine |
| Diario migraña | Spanish | Net Workz LLC |
| Dolor crónico | Spanish | LetltGuide SL. |
| Dolor de cabeza-Diario/calendario/registro | Spanish | TrinnVis as |
| Dor de Cabeça / Headache App | English, Portuguese | SODA |
| El dolor de espalda | Spanish | Cecile Maurech |
| Estiramientos | Spanish | Ana Gallardo |
| Fitness at Work | Chinese, English | Tusitala Pte Ltd |
| Get rid of a toothache naturally in less than 12 hours | English | Reimund Lube |
| Headache Diary | English, French, German, Japanese, Spanish, Swiss | Jae-Ung Yi  John Seok M.D. |
| Headache helper | English | Bell Standard, Inc. |
| Headache Tracker | English | Joseph Southern |
| Headache Wiper | English | BeMor Mobile Pty Ltd |
| Headaches | English | S Liu |
| Headaches | English | TENRAYsOFT, LLC |
| i Ankylosing  Spondylitis | English | Anatomate-Apps |
| iHeadache Headache & Migraine Diary | English | BetterQOL, Inc. |
| IREHAB Back pain | Japanese, Spanish | iREHAB.com LLC |
| iSleepAid | English | Vanilla Breeze Co.,Ltd |
| Knee Pain | English | Ortho Tech Inc |
| Kyusho Healing | English | Interactive Motion |
| Manage IBS now | English | Tommy Connolly  David Ridgeway |
| Migraine stop | English | TENRAYsOFT, LLC |
| Migraine Tracker! | English | Sage Young |
| Miniatlas del dolor | English, Spanish | Licitelco S.L |
| Miniatlas del dolor neuropático | English, Spanish | Licitelco S.L. |
| Miniatlas sistema nervioso central | Spanish | Licitelco S.L. |
| My best medicine | English, French, German, Japanese, Korean, Spanish | Jae-Ung Yi |
| myIBD | English | Toronto’s Hospital for Sick Children, Canada |
| My Pain Diary | English | Damon Lynn, USA |
| MyoCycle | English | MyoMedic PTY Ltd |
| Odontalgia Pro | Spanish | DtD BV |
| OIDometer | Spanish | Entorno Digital de Comunicación SL |
| Opioides | Spanish | Grünenthal de Mexico SA de CV |
| Opioids Dosage Conversion | English | Santa Clara Valley Medical Center & Chris Marcellino |
| Pain | English | Doctot |
| Pain Care | English | Ringful Health, Robert Wood Johnson Foundation |
| Pain diary | English, French, German, Japanese, Spanish, Swiss | Jae-Ung Yi  John Seok M.D. |
| Pain therapy- Terapia de dolor | Spanish, English subtitles | Winzig |
| Pain Relieve English | English | Hans-Peter Zimmermann |
| Pauseboogie | Danish | Plant ApS, Denmark |
| Phrase Board | Arabic, English, French, German, Italian, Spanish, Swedish | Eamonn and Ian LLC, USA |
| Prevent Back & Spine Pain | English | iGlimpse Ltd |
| Prevent Lower Back Pain | English | iGlimpse Ltd |
| Prevent Upper Back & Neck Pain | English | iGlimpse Ltd |
| Release Pain with Andrew Johnson | English | Michael Schneider |
| Rehuma Track | English, French, German | Mutterelbe Medical Ug |
| Shoulder Injuries | English | Emanuel Petroulakis |
| Shoulder Pain | English | Ortho Tech Inc |
| SmallTalk Intensive Care | English | Lingraphicare America Inc, USA |
| SmallTalk Pain Scale | English | Lingraphicare America Inc, USA |
| SOSdolm | Spanish | ExtremeDevelopment S.L |
| The Simplyhealth Back Care app | English | FivebyFive, UK |
| Therappee | English | Huntenhull |
| Tip Share | English, Spanish | ConRadical LLC, USA |
| Therapy Diary | English | Jae-Ung Yi |
| Traductor de síntomas de salud en 18 idiomas | Catalan, English, Spanish | COFB64 SERVEIS SL |
| Tummy tracker | English | Tolemy communications Pty limited |
| Universal-Yoga for Migraines | English | Saagara LLC |
| VAS translator | Arabic, English, French, Spanish, Portuguese, German, Polish, Serbian, Bulgarian, Romanian, Norwegian, Swedish, Danish, Filipino, Moroccan, Turkish, Chinese | Geerten van Hooff |
| Village Dental Care | English | Mike Litman |
| Virtual Doctor | English | DSHI Systems, Inc |
| WebMD | English | WebMD LLC |
| WebMD Pain Coach | English | WebMD LLC |
| Yoga for Migraines | English | Saagara LLC |
| Zen relax | Spanish | Avant Wb Solutions SL |

**S1.B Blackberry World (Blackberry)**

| **App Name** | **Language** | **Developer** |
| --- | --- | --- |
| A-Z Muscle Trigger Points | English | Vital Acts Inc. |
| Alleviate Migraines For BlackBerry 10 | English | Paul Mackinnon |
| Acupressure Points for Headaches and Migraines | English | MANYA Technologies |
| Acupressure: Treat Yourself | English, Spanish | AM Mobile |
| Back Pain Relief | English | bigo |
| Brain Sooth | English | EMOBISTUDIO |
| Cómo Aliviar los Dolores de Espalda | Spanish | TappCoder |
| Cure back pain through yoga | English | Winjit Technologies Pvt Ltd |
| Foot Pain | English | LA Store |
| Headache Diary | German | Schroederonline IT |
| Headache History | English | Kopong Mobile |
| iHeadache Headache & Migraine Diary | English | BetterQOL, Inc. |
| Massage Deck | English | Mobifusion Inc |
| Medicine Cabinet Head | English, German | EBS Germany |
| Muscle Trigger Points Doctor | English | Vital Acts Inc. |
| Neck and shoulder pain quiz | English | songfei |
| Pain Manager | English | Vimukti Technologies Pvt Ltd |
| PlayCoach™ Fitness Back Pain | French | Playcorp |
| Prevention from headache | English | AKSHAY JAIN |
| Reflexology | English | Popup Technologies.com |
| Sota Omoigui's Pain Drugs Handbook (paindrugs) | English | Skyscape, Inc. |
| Where Does It Hurt - LITE | English | K2 Media and Web Design Inc. |

**S1.C Google Play (Android)**

| **App Name** | **Language** | **Developer** |
| --- | --- | --- |
| 250 Pain With Treatment | English | Uniqueapp |
| Acuapp | English | Maitri Hillebrecht |
| Acupresión Dolor De Cabeza | English, Italian, French, German, Norwegian, Russian, Spanish | Alchemy |
| Acupressure Treat Yourself | English | Alexander Mokrushin |
| Acupuncture For Aches | English | Appzdepo |
| Aliviar Los Dolores De Espalda | Spanish | Tap Coder |
| Analgesia Práctica | Spanish | Upcb Apps |
| Aptitud Del Dolor De Espalda | French | Playcorp |
| Arthritis Joint Pain Handbook | English | Dhruvi |
| Arthritis Joint Pain Relief | English | Koolappz |
| Artrosis | Spanish | Pcr Aurope |
| Aspirina Virtual | Spanish | Giovanni G |
| Avoiding Back Pain | English | Quality Mrrsitd |
| Ayurveda Cures Remedies | English | Arpita Suroshe |
| Back Exercises | English | Bigo |
| Back Pain | English | Bawidgets.Com |
| Back Pain | English | Nic |
| Back Pain | English | Ortho Tech Inc |
| Back Pain Diagnosis | English | MatheMEDics, Inc |
| Back Pain Guide | English | Wonderworlapps |
| Back Pain Guides | English | Renu Jain |
| Back Pain Massager | English | Apps Zenny |
| Back Pain Relief | English | Bigo |
| Back Pain Relief | English | Mordevz |
| Back Pain Relief  (Backpain The Natural Way) | Spanish | Arweebapps |
| Back Pain Relief Guide | English | App Maniac |
| Back Pain Relief Guide 2013 | English | Padhiyar |
| Back Pain Relief- Free | English | Better Health |
| Back Pain Remedies | English | Joliallc |
| Back Pain Solutions/Remedies | English | App Maniac |
| Back Pain Symptoms & Treatment | English | Bannerdock, Inc |
| Bcx Eva Escala Dolor | Spanish | Biocapax Technologies |
| Best Symptom Checker | English | Senstore-Powered By Harvard |
| Body Pain Management Hypnosis | English | Grand Apps Studio |
| Chest Pain Diagnosis | English | MatheMEDics, Inc |
| Chronic Pain Connects | English | Alliance Health Networks |
| Comprehensive Medicine | English | Loyal Programs |
| Dealing With Back Pain | English | Book App |
| Dealing With Back Pain | English | Koolappz |
| Diagnosaurus Ddx | English | Unbound Medicine,Inc  Roni Zeiger |
| Diario De Dolor De Cabeza | Spanish | Benjamin Gerfelder |
| Dienchan Facy Therapy | English | Viet Y Dao Bui Quoc Chau |
| Doleur | French | Blevruz |
| Dolor Crónico | Spanish | Letitguide Sl |
| Dolor De Cabeza Wellwave | English | Alchemy |
| Dolor De Espalda | Spanish | Iappodium Labs |
| Escala De Dolor | English, Spanish | Trate A Dor |
| Escalas Médicas | Spanish | Dedihealth |
| Estiramientos | Spanish | Be On Marketing Online |
| FibroMapp | English | Bodymap Apps |
| Fibromyalgia | English | Guides |
| Fibromyalgia Magazine | English | Magazinecloner.Com |
| Fisioterapia y Dolor | Spanish | J Caberizo Hospital Universitario Henares |
| Fix Pain Hypnosis | English | Grand Apps Studio |
| Hand Massage Acupressure | English | Shapes |
| Headache | English | Guides |
| Headache 101 | English | Ls Apps Dev |
| Headache App | English | Postano |
| Headache Diagnosis | English | MatheMEDics, Inc |
| Headache Diary | English | Marcel Schroder |
| Headache Diary | English | Froggyware |
| Health And Beauty Part 1- Free | English | Appbuzz |
| Herniated Back Pain Relief Now | English | Primoapps |
| How To Cure Lower Back Pain | English | Appsforbusinessgeek |
| How To Cure Tmj | English | Bigo |
| Irritable Bowel Syndrome | English | Vertex Mind Llc |
| Itriage Elath | English | Itriage, Llc |
| Jaquecapp | Spanish | Terranology |
| Joint Pain Relief Now | English | Dietsandfads |
| Kendall Pain Relief Center | English | Cloud Nine Development |
| Knee Pain | English | Ortho Tech Inc |
| Knee Pain Relief | English | Easysource |
| Lebanon Pain Relief Center | English | Cloud Nine Development |
| Left Side Lower Back Pain | English | Softomatrix |
| Live Pain Free The Natural Way | English | Apps Ahoy |
| Living With Crohn´S Disease | English | Vertex Mind Llc |
| Living With Fibromyalgia Pv | English | Vertex Mind Llc |
| Manage My Pain | English | Managinglife |
| Medical Spanish By Mavro | English  Spanish | Mavro Inc |
| Meditation Anywhere Pain | English | Meditationanywhere |
| Miniatlas Dolor | Spanish | Clyna S.A |
| Miniatlas Dolor Crónico | Spanish | Clyna S.A |
| Miniatlas Dolor Neuropático | Spanish | Clyna S.A |
| myIBD | English | Toronto’s Hospital for Sick Children, Canada |
| Muscle Trigger Point Anatomy | English | Real Bodywork |
| Natural Arthritis Relief | English | Appzdepo |
| Natural Pain Management | English | Vertex Mind Llc |
| Natural Pain Management | English | Biz App Media |
| Natural Pain Management | English | Paddleo |
| Natural Pain Management | English | Koolappz |
| Natural Pain Remedies | English | Selvi M |
| Nature Sound | English | Zodinplex |
| Nccp Breast Pain Diary | English | Cpd Sessions |
| Neck Pain Prevention | English | Templebellchime |
| Neck Pain Relief | English | Easysource |
| No Back Pain Problems | English | Umeetone |
| Nurse´s Drug Guide 2011 Tr | English | Mobile Systems, Inc |
| Oidometer | Spanish | Entorn Digital De Comunicació |
| Online Anesthesia | English  Spanish | Arjus Limited |
| Orthopedic Pain Management | English | It Mentor Apps |
| Pain | English | Jere Parker |
| Pain & Depression/Ambiscience | English | Tesla Software, Llc |
| Pain Acupressure | English | Jeejee |
| Pain Assessment | English | Kuntec |
| Pain Audiobook | English | Twayesh Projects |
| Pain Care | English | Ringful Health, Robert Wood Johnson Foundation |
| Pain Control- R Collin | English | I-Mobilize Inc  Rick Collingwood |
| Pain Easer | English | Jeejee |
| Pain Log | English | Kshitij Grover |
| Pain Management Pocket | English | Borm Bruckmeier Publishing Llc |
| Pain Release Hypnosis Pr | English | Awake Media Publications |
| Pain Relief | English | Guides |
| Pain Relief | English | Obstetric Anesthetists Association |
| Pain Relief 2.0 | English | Zeleniak |
| Pain Scale | English | Guo Jhong-Yi |
| Pain Treatment | English | Wait |
| Painometer V2 | Catalan, English, French, Portuguese, Spanish | ALGOS-Research on Pain |
| Pauseboogie | Danish | Plant ApS, Denmark |
| Pediatric Pain Journal V2 | English | Greggh |
| Perfect Posture Exercises | English | Tommy Tessandori |
| Physiotherapy Back Pain | English | Moreapps |
| Prevent Back & Spine Pain | English | iGlimpse Ltd |
| Prevent Lower Back Pain | English | iGlimpse Ltd |
| Prevent Upper Back & Neck Pain | English | iGlimpse Ltd |
| Rehuma Track | English, French, German | Mutterelbe Medical Ug |
| Relieve Sciatic Nerve Pain | English | Candiapp |
| Rückenschmerz Manager Lite | German | Julia Bechmann |
| Sciatica Relief | English | Bigo |
| Scores Pediatría | Spanish | Joselu |
| Shoulder Pain | English | Ortho Tech Inc |
| Shoulder Pain Treatment | English | Trafficapps |
| Simplyhealth Back Care App | English | Fivebyfive |
| Síntomas De Salud | Catalan, English, Spanish | Farmaguía |
| Sleep Hypnosis-Back Pain | English | Sleep Learning |
| Solutions Of Back Pain | English | Dsrsol |
| Symptom Based Radiology | English | Symptom Based Radiology |
| The Pain App | English | Social Care Alba |
| The Simplyhealth Back Care app | English | FivebyFive, UK |
| Tip Share | English, Spanish | ConRadical LLC, USA |
| Universal Yoga For Back Pain | English | Saagara |
| Upper& Lower Back Pain | English | Nicholas Gabriel |
| WebMD | English | WebMD LLC |
| WebMD Pain Coach | English | WebMD LLC |
| Wellwave Carpal Tunnel | English | Alchemy |
| Wellwave | English | Alchemy |
| Wellwave Arthritis | English | Alchemy |
| Wellwave Back Ache | English | Alchemy |
| Wellwave Painkill | English | Alchemy |
| Wellwave Tooth Ache | English | Alchemy |
| What Causes Back Pain? | English | Zingy Minds |
| Where Does It Hurt | English | K2 Media And Web Design Inc |
| Yoga For Back Pain | English | Saagara |

**S1.D Nokia (Ovi)**

| **App Name** | **Language** | **Developer** |
| --- | --- | --- |
| 101 Prevent Headache Tips | English | Chourishi |
| Acupressure Treat Yourself | English | Alexander Mokrushin |
| Female Sex Problems - Sex Pain | English | GizmoBuddy |
| Massage Deck | English | Mobifusion Inc |
| Massager (Asha) | English | Vishnu_a93 |
| Menstruation and Ovulation | English | EFRAC |
| Plantar Fasciitis | English | CannyTech |
| Tuotromedico | Spanish | J1CK |

**S1.E Windows phone**

| **App Name** | **Language** | **Developer** |
| --- | --- | --- |
| Acupressure: Treat Yourself | German, English, French, Italian, Norwegian, Russian, Spanish | AM Mobile |
| Arthritis Relief | English | KoolApz |
| Back Pain Guide | English | KoolApz |
| Chiropractic Care | English | KoolAPz |
| Dr Nature Emergencias | Spanish | joserafael1990 |
| Fibromyalgia | English | Hypnotransformations LLC |
| Foot Reflexology | English | pedromeca |
| Headaches | Spanish | Sergio Cantos Maqueda |
| Living with arthritis | English | KoolApz |
| Natural Arthritis Relief | English | KoolApz |
| Pain evaluation in children | English | Andrea Ciarrochi |
| Pain Trucker Plus | English | HealthSaaS Inc |
| PainDiary | English | Andrew Brooks |
| Relief Neck Pain | English | Tak Wai Wong |
| Relief Shoulder Pain | English | Tak Wai Wong |
| Relief Wrist Pain | English | Tak Wai Wong |
| Scores Pediatría/ Pedriatic Scores | English, Spanish | Joselu |
| The Pain Reporter Lite | English | Dvimay Technologies |
| Treating Headaches | English | KoolApz |

**Note:** This list contains the apps retrieved using the search terms “pain”, “ache” and “dolor” for all of the shops.
