# Supplementary material for: mHealth: A Strategic Field without a Solid Scientific Soul. A Systematic Review of Pain-Related Apps
Source: PLoS One. 2014 Jul 7;9(7):e101312. doi: 10.1371/journal.pone.0101312 (PMC4085095; doi:10.1371/journal.pone.0101312)
Supplement: Table S2 — Characteristics of the commercial apps that have some sort of support. (DOCX) [file pone.0101312.s002.docx]

**Table S2. Characteristics of the commercial apps that have some sort of support**

| **App name** | **Developer** | **Support** | **Pain problem** | **Features** | **Platform and price** | **Language/s** | **Users rating** | **Description as provided by the developers** |
| --- | --- | --- | --- | --- | --- | --- | --- | --- |
| Afa-MICI | Franck Touboul, Ylly, France | Created by:  Association François Aupetit (AFA), France | Inflammatory bowel disease (IBD), | Allows networking with other patients / Family / Friends  Information | Apple  Free | Czech  Dutch  English  French  German  Italian  Japanese  Korean  Polish  Portuguese  Russian  Simplified Chinese  Spanish  Swedish  Traditional Chinese  Turkish | 5/5^a^ | Provides information on inflammatory bowel disease (IBD), dieticians and hospitals, and on the activities of France-based patient group, Association Francois Aupetit (AFA). Contains a lavatory finder, allowing the user to also input the location of facilities not on the existing database. Provides access to AFA forum posts. |
| Aps SmartCal | Meed Comunicación SL, Spain | Created by:  Dr. Raquel Almodóvar, MD  Rheumatology service, “Fundación Alcorcón” Hospital | Psoriatic Arthritis | Information  Patient monitoring | Apple  Free | Spanish | 5/5 | This application put together several indices for the assessment of psoriatic arthritis: PASI, DAS28, BASDAI, BASFI, pain scales and HAQ-20 to offer the rheumatologist a tool to track the overall evolution of the patient over time. |
| ArthritisID | ACE Planning and Consulting Inc, Canada | Created by:  Arthritis Consumer Experts (ACE), Canada  Recommended by:  Arthritis New Zealand  Canadian Spondylitis Association  A Group Representing patients who suffer from Rheumatological problems, Canada | Some arthritis types:  Ankylosing Spondylitis, Gout, Juvenile Idiopathic Arthritis, Lupus, Osteoarthritis, Psoriatic Arthritis, and Rheumatoid Arthritis. | Information  Support to deal with symptoms/disabilities | Apple  Browser  Free | English  French | 4.5/5^a^ | Contains information on detecting, treating and managing arthritis. Information on the prevention of arthritis includes facts about exercise, diet and nutrition. Treatment strategies and details about medication are discussed. An interactive arthritis screening tool and questionnaire helps the user detect indications of arthritis. Personal information about arthritis can be saved. |
| Back Pain | Dr. Gozna, Canada  Ortho Tech Inc | Created by:  Dr. Eric Gozna, MD, Orthopedic Surgeon, Professional Biomedical Engineer | Low Back Pain | Information | Android  2·15$  1·56€  Apple  8·23$  5·99€ | English | Not rated | A course for both healthcare professionals and patients. In easily understandable language it explains current concepts regarding its cause and common contributors. It also presents a systematic approach to its diagnosis and management. |
| Back Pain Diagnosis | MatheMEDics, Inc, USA | Created by:  Martin F. Sturman, MD, FACP  Henry R. Sturman, M.Sc.Eng  Richard N. Harner, M.D.  Manuel Perez, Ph.D.  Paul J. Rosch, MD, FACP  Jack Sizer, M.D., MBA  Julie Brown Sturman, BFA, RN  M. Elayne DeSimone, Ph.D., NP-C  Joel M. Mynders, A.B. BC-HIS | Back Pain | Diagnosis | Android  1·32$  0·69€ | English | 5/5 | By asking a series of questions about the back pain, the expert system will analyze the answers and provide a list of the most likely diagnoses in order of probability. |
| Bcx Eva Escala Dolor | Biocapax Technologies, Spain | Created by:  Nursing council of the Valencian community | Pain | Patient monitoring | Android  Free | Spanish | 4.3/5 | It is intended for use as support for the daily activities of health care professionals and family caregivers who want to assess pain analogically. |
| Best Android Symptom Checker | Senstore, USA | Created by:  Harvard Medical School | Various types of pain: abdominal, chest, headache, etc. | Medical advice | Android  Free | English | 4.6/5 | Symptom checker which helps patients answer the question: "Do I need to see the doctor?" |
| Body Pain Management Hypnosis | Grand Apps Studio, USA | Created by:  Elizabeth Harford, licensed UCM minister and certified hypnotherapist by the "The National Guild Of Hypnotists" | Pain intensity | Support to deal with symptoms/disabilities | Android  Free | English | 3.8/5 | Hypnosis audio |
| Chest Pain Diagnosis | MatheMEDics, Inc, USA | Created by:  Same as previous entry | Chest Pain | Diagnosis | Android  1·32$  0·69€ | English | 5/5 | By asking a series of questions about chest pain, the expert system will analyze the answers and provide a list of the most likely diagnoses in order of probability. |
| Diagnosaurus Ddx | Unbound Medicine, USA | Created by:  Roni F. Zeiger, MD  Published by:  McGraw-Hill professional | Various types of pain: abdominal, chest, headache, etc. | Diagnosis | Apple  2·46$  1·79€ | English  French  Simplified Chinese | Not rated | A quick-reference tool developed to help healthcare professionals perform differential diagnosis with speed and confidence at the point of care. |
| Dr. Nature EMERGENCIAS | joserafael1990, Mexico | Created by:  Dr. Rafael García Chacón, President of the Association of Integrative Medicine AMHI | Various types of pain | Support to deal with symptoms/disabilities | Windows Phone  Free | Spanish | Not rated | This application finds natural solutions to the home emergencies we all have such as colic or a toothache at 3 in the morning. |
| FibroMapp | BodyMap Apps, UK | Created by:  Fibromyalgia Association (FMA) UK | Fibromyalgia | Information  Reminders  Self-monitoring  Support to deal with symptoms/disabilities  Trackers | Android  6·81$  5·09€ | English | 4.5/5^a^ | This app is a sleep tracker, a symptoms tracker, a medications tracker, and an alarm system. It is also a journal, in which the user can log the severity of pain, the location of pain, and the type of pain. The app has charting, a report-writing facility, and allows printout and email. |
| Headache Diagnosis | MatheMEDics, Inc, USA | Created by:  Same as the previous one | Headache | Diagnosis | Android  1·32$  0·69€ | English | 5/5 | By asking a series of questions about the headache, the expert system will analyze the answers and provide a list of the most likely diagnoses in order of probability. |
| Headache Wiper | BeMor Mobile, Australia | Created by:  Des Morrow, Physical Therapist. Director of the Australian Spine Institute. | Headache | Support to deal with symptoms/disabilities | Apple  4·99$  3·63€  Free (Lite) | English | Not rated | The PRO App contains 8 easy to follow short videos showing specific exercises and stretches aimed at relieving headache and preventing future ones. |
| iAnkylosing  Spondylitis | Anatomate-Apps, Australia | Recommended by:  Arthritis New Zealand | Ankylosing spondylitis | Information  Self-monitoring  Support to deal with symptoms/disabilities | Apple  Free | English | 4.5/5^a^ | Educational app. Contains voice and text videos that introduce the condition’s symptoms, and refer to associated diseases, diagnostic criteria, genetics, incidence, morbidity/mortality, pharmacological management, and physical therapy. Includes medical imaging, showing sacro-iliitis and syndesmophytes, treatment suggestions and exercise animations. |
| iHeadache | Better QOL, USA | Created by:  Brian D. Loftus, MD  Recommended by:  National Headache Foundation (NHF), USA | Headaches | Self-monitoring  Support to deal with symptoms/disabilities  Trackers | Apple  4·84$  3·76€  'Lite' free  Blackberry  4·99$  3·96€ | English | 3.25/5^a^ | Headache diary that tracks the number of headaches, their duration and severity, the degree of disability and impact, medication and usage, symptoms, and triggers. It can generate reports to be shared with a doctor. |
| Knee Pain | Dr. Gozna, Canada  Ortho Tech Inc | Created by:  Dr. Eric Gozna, MD, Orthopedic Surgeon, Professional Biomedical Engineer | Knee Pain | Information | Android  2·14$  1·56€  Apple  8·23$  5·99€ | English | Not rated | In easily understandable language this course explains the common causes, diagnosis and management of knee pain.  Intended for healthcare providers and patients. |
| Manage My Pain | Life Control Software Inc/ManagingLife, Canada | Recommended by:  Slovak League Against Rheumatism | Pain | Self-monitoring  Support to deal with symptoms/disabilities  Trackers | Android  4·83$  3·53€ | English  Russian | 3.25/5^a^ | Allows a user to track and record pain on a customizable entry screen. Displays the information in statistics and graphs. |
| My Pain Diary | Damon Lynn, USA | Created by: RSD/CRPS patient  Recommended by:  World Pain Foundation (WPF)  Awards:  Best App Ever Awards 2013 Winter/Spring 2012 Web Health Award | Allergies, back pain, chronic fatigue syndrome (CFS), depression, fibromyalgia, headaches, or migraine. | Reminders  Self-monitoring | Apple  9·99$  7·27€ | English | 4.5/5^a^ | Intensity of pain, its locations, types, triggers, and treatments can be documented. Up to three text notes can be saved. Interactive graphs help in finding correlations between factors and the weather. Reminders to keep entries consistent can be issued. Password protected. Data can be backed up via ‘Dropbox’. |
| myIBD | Toronto’s Hospital for Sick Children, Canada | Created by:  Karen Frost,  Dr. Johan Van Limbergen  Meaghan Wright  Ritchie Hwang  Recommended by:  Crohn’s and Colitis Australia  Collaborative Chronic Care Network, USA | Inflammatory bowel disease (IBD) | Information  Self-monitoring  Support to deal with symptoms/disabilities  Trackers | Android  Apple  Browser  Free | English | 4.5/5^a^ | Aims to provide the user with new perspectives on the task of managing inflammatory bowel disease (IBD). Allows the recording of important personal healthcare information, including appetite, mood, pain, and visits to the lavatory. Entries can be viewed as a graph, or as text. The app also contains information about IBD. |
| Opioids Dosage Conversion | Santa Clara Valley Medical Center & Chris Marcellino, USA | Created by:  Norris Vivatrat, MD | Pain | Support to opiod dosage | Apple  Free | English | 4.5/5 | Opioid analgesic dosage conversion calculator for use by trained medical professionals. |
| Pain | Doctot, Ireland | Created by:  Declan Lyons Professor of Medical Science & Consultant Physician at University Hospital Limerick | Pain | Patient monitoring  Support to opiod dosage | Apple  7·55  5·49€ | English | Not rated | The app includes the most widely used clinician-administered assessment scales and lookup tables related to Pain patients. It contains an opioid equivalence comparison lookup. |
| Pain Assessment | Kuntec, UK | Created by:  University of Greenwich  British Geriatrics Society  The British Pain Society | Pain | Patient monitoring | Android  Free | English | Not rated | Assessment of pain in older people. |
| Pain Care | Ringful Health, USA | Created by:  Dr Vishal Kancherla  Recommended by:  Family Caregiver Alliance (FCA), USA  Louise H. Batz Patient Safety Foundation, USA | Chronic pain or sports injuries | Information  Reminders  Self-monitoring  Support to deal with symptoms/disabilities  Trackers | Android  Apple  Free | English | 4.5/5^a^ | Allows the user to track medications, side effects, symptoms of pain, and triggers. After the journal has been kept for some time, the app will estimate the cause of the pain, and consider the efficacy of treatment/medication options. Patient history, with analytic charts designed to fit into the decision-making workflow of pain specialists. Data can be shared with a doctor securely via the Internet. |
| Pain Relief | Obstetric Anaesthetists' Association, UK | Created by:  Obstetric Anaesthetists' Association | Labour and Delivery | Support to deal with symptoms/disabilities  Information | Android  Free | 35 languages | 4.6/5 | This app is aimed at mothers and their partners, as well as health care professionals.  Pain Relief in Labour; Anesthesia for Caesarean delivery; Epidural information; Useful phrases (phonetic) and High Body Mass Index |
| Painometer V2 | ALGOS. Research on Pain, Spain | Created by:  ALGOS. Research on Pain (Universitat Rovira i Virgili)  Awards:  Mobile World Capital mHealth Award | Pain intensity | Patient monitoring  Self-monitoring | Android  Browser  Free | Catalan  English  French  Portuguese  Spanish | 4.8/5 | Contains four well-known and validated pain intensity scales. Users can save pain intensity data, as well as information about the scale used, the day and the time of assessment. Besides, one can see the evolution of the pain intensity in a graph, and send the data to an email address. |
| Pauseboogie | Plant ApS, Denmark | Created by:  Gigtforeningen (patient organisation specialising in arthritis) Denmark | Arthritis or osteoarthritis | Information  Support to deal with symptoms/disabilities | Android  Apple  Browser  Free | Danish | 4.5/5^a^ | Demonstrates how to perform a series of exercises intended to ease and prevent pain and discomfort in muscles and joints. Lists exercises for the back, feet, hips, knees, neck, and shoulders. |
| Phrase Board | Eamonn and Ian LLC, USA | Recommended by:  Central Coast Children Foundation, USA  Patient group specializing in communication disability, Australia | Patients with speech difficulties | Improving communication  Support to deal with symptoms/disabilities | Apple  Free (English)  The other six languages are available through in-app purchase | Arabic  English  French  German  Italian  Spanish  Swedish | 2.25/5^a^ | Allows people who cannot speak to display some basic concepts to other people. Large ‘Yes’ and ‘No’ buttons offer a means of simple expression. Scrollable lists of ‘Having’, ‘Feeling’, and ‘Want’ statements provide for quick communication. Custom messages and frequently employed phrases can be input and saved. A message bar highlights statements. A ‘Pain’ page allows pain to be expressed. A slider displays pain on a one-to-ten scale. Duration, frequency, location and type of pain can all be expressed. |
| Prevent Back & Spine Pain | iGlimpse Ltd, UK | Created by:  Dr Philip Striano, certified chiropractic sports physician | Back Pain | Support to deal with symptoms/disabilities | Android  3·42$  2·49€  Apple  1·99$  1·45€ | English | 5/5 | This app helps patients to learn a variety of exercises that stretch, strengthen, and stabilize important muscles and ligaments supporting the spine and core. |
| Prevent Lower Back Pain | iGlimpse Ltd, UK | Created by:  Dr Philip Striano, certified chiropractic sports physician | Lower Back Pain | Support to deal with symptoms/disabilities | Android  3·43$  2·50€  Apple  1·99$  1·45€ | English | 5/5 | This app helps patients to learn a variety of exercises that stretch, strengthen, and stabilize important muscles and ligaments that support the lower back. |
| Prevent Upper Back & Neck Pain | iGlimpse Ltd, UK | Created by:  Dr Philip Striano, certified chiropractic sports physician | Upper Back and Neck Pain | Support to deal with symptoms/disabilities | Android  3·43$  2·50€  Apple  1·99$  1·45€ | English | 5/5 | This app helps patients to learn a variety of exercises that stretch, strengthen, and stabilize important muscles and ligaments that support the neck and back. |
| Rheuma Track | Nicole Derouaux of Mutterelbe Medical UG, Germany | Created by:  Dr. Peer Malte Aries, Rheumatologie, Klinische Immunologie, Hamburg, Germany/EULAR | Arthritis and  ankylosing spondylitis | Information  Reminders  Self-monitoring  Support to deal with symptoms/disabilities  Trackers | Android  Apple  Browser  Free | English  French  German | 4.5/5^a^ | Helps people with rheumatism monitor their condition. Personal facts (for example, inability to work, morning stiffness, times of infection, etc) can be recorded and tracked. Functional-ability assessment questionnaire of symptoms. Pain levels can be logged in a ‘Pain diary’ (‘Visual Analogue Scale’, VAS). Represents rheumatism by means of a tracing development, lists, or calendars. Notes can be taken. Issues reminders of due dates to collect replacement medications. |
| Shoulder Pain | Dr. Gozna, Canada  Ortho Tech Inc | Created by:  Dr. Eric Gozna, MD, Orthopedic Surgeon, Professional Biomedical Engineer | Shoulder Pain | Information | Android  5·13$  3·73€  Apple  8·23$  5·99€ | English | Not rated | This course explains the 6 most common causes of shoulder pain, how they are diagnosed/treated and shows a simple 3-minute examination to identify each one.  Intended for healthcare providers and patients. |
| SmallTalk Intensive Care | Lingraphicare America Inc, USA | Recommended by:  Central Coast Children Foundation, USA  Aphasia Hope Foundation (AHF), USA | Patients with speech difficulties | Improving communication  Support to deal with symptoms/disabilities | Apple  Browser  Free | English | 3.5/5^a^ | Provides a list of phrases and images with which patients can communicate to medical professionals some of their needs and feelings (such as "I have chest pain", or "I want a doctor"). Intended for patients and people who have difficulty speaking and for people who cannot speak at all. Phrases chosen are spoken in a natural human voice, and accompanying pictures emphasise meaning. |
| SmallTalk Pain Scale | Lingraphicare America Inc, USA | Recommended by:  Central Coast Children Foundation, USA  Patient group specialising in communication disability, Australia | Patients with speech difficulties | Improving communication  Support to deal with symptoms/disabilities | Apple  Free | English | 4.5/5^a^ | Allows people who cannot speak (particularly people with aphasia, apraxia, or dysarthria) to communicate amount and type of pain felt to other people (including caregivers, family members, and healthcare professionals). Provides a vocabulary of pictures that talk in a natural human voice. The app’s pain scale is based on images and descriptions of pain from the ‘Wong-Baker Faces Pain Scale’. |
| The Simplyhealth Back Care app | FivebyFive, UK | Created by: Simplyhealth (Healthcare provider)  In collaboration with:  BackCare (Charity) and Nuffield Health (not for profit organization) | Back Pain | Information  Support to deal with symptoms/disabilities | Android  Apple  Free | English | 3.6/5 | It offers health information and advice to help manage and prevent back pain. There are graphics and videos of exercises and allows patients to keep a pain diary. |
| Therappee | Huntenhull, UK | Created by:  Tim Allardyce (MCSP, SRP)  Professional sports physiotherapist | Pain | Information  Support to deal with symptoms/disabilities | Apple  Free (it allows in-app purchase) | English | Not rated | The app includes some exercise videos designed to help relieve any sort of pain. |
| Tip Share | ConRadical LLC, USA | Created by:  Arthritis Foundation (patient group specializing in arthritis)  Recommended by:  Community Health Charities of the National Capital Area USA | Arthritis | Information  Support to deal with symptoms/disabilities | Android  Apple  Browser  Free | English  Spanish | 4.5/5^a^ | Helps the user to manage joint pain from arthritis by reading advice and ‘tips’ from other people who are living with the condition. The user can share personal tips about managing arthritis pain, rate other people’s tips, and view tips supplied by the Arthritis Foundation. |
| WebMD | WebMD LLC, USA | Created by:  WebMD LLC, USA  (Certified website)  Recommended by:  National Alliance on Mental Illness (NAMI) Lane County, USA | Various health conditions | Information  Support to deal with symptoms/disabilities | Android  Apple  Free | English | 4.5/5^a^ | Contains informational tools that can guide a user in making decisions about personal health. A symptom checker aids in identifying potential conditions or health issues. Medically-reviewed information about conditions is available. Contains a database on drugs, supplements, and vitamins, with details about usage, side effects, warnings, etc. |
| WebMD Pain Coach | WebMD LLC, USA | Created by:  WebMD LLC, USA  (Certified website) | Chronic pain conditions: Back pain  Neck pain  Nerve pain  Fibromyalgia  Migraine  Osteoarthritis  Rheumatoid arthritis | Information  Reminders  Self-monitoring  Support to deal with symptoms/disabilities  Trackers | Android  Apple  Free | English | 4/5 | The app offers a holistic approach to balancing lifestyle with chronic pain conditions to help inspire a better day. |

^a^ This rating was extracted from “http://myhealthapps.ne”. It assesses how well the app does the following: “helps you control your condition/keep you healthy, Is trustworthy, Is easy for you to use, Allows you to network with people like you/who understand you, Can be used regularly”. All other ratings were extracted from Android and Apple users’ opinions.

PASI: Psoriasis Area Severity Index, DAS28: Disease Activity Score, BASDAI: Bath Ankylosing Spondylitis Disease Activity Index, BASFI: Bath Ankylosing Spondylitis Functional Index, HAQ-20: The Stanford Health Assessment Questionnaire.
