# Supplementary material for: mHealth: A Strategic Field without a Solid Scientific Soul. A Systematic Review of Pain-Related Apps
Source: PLoS One. 2014 Jul 7;9(7):e101312. doi: 10.1371/journal.pone.0101312 (PMC4085095; doi:10.1371/journal.pone.0101312)
Supplement: Protocol S1 — Protocol for the systematic review. (DOCX) [file pone.0101312.s003.docx]

**S3. Protocol for the systematic review**

**S3.A Review of pain related applications reported on scientific databases**

Search strategy and selection criteria

Preferred Reporting Items for Systematic Reviews and Meta-Analyses (PRISMA) guidelines were followed.

Data for this review were identified by searches of the following databases: Medline (National Library of Medicine), Science Citation Index Expanded (Web of Science), Health Reference Center Academic (Gale), Wiley Online Library, American Psychological Association (Psycinfo), SciVerse ScienceDirect (Elsevier), SpringerLink, Wolters Kluwer - Ovid - Lippincott Williams & Wilkins (CrossRef), Directory of Open Access Journals (DOAJ), Social Sciences Citation Index (Web of Science), Taylor & Francis Online - Journals, Expert Reviews (Future Science), Informa - Informa Healthcare (CrossRef), SpringerLink Open Access, Wolters Kluwer - Ovid (CrossRef), BMJ Journals, DiVA - Academic Archive Online, Informa (CrossRef), and references from relevant articles.

The search terms were: (Pain OR *ache) AND (Smartphone OR app OR application OR electronic OR “Personal Digital Assistant” OR PDA).

Only peer-reviewed articles published in English or Spanish between 1996 and December 2013 were included.

**S3.B Review of pain related applications available in the main stores**

In December 2013, the main Smartphone application shops were reviewed: App Store (iPhone), Blackberry App World, Google Play (Android), Nokia Store and Windows Phone Store.

The review was conducted in the following countries: Canada, Spain, and USA.

The search terms were: “Pain”, “*ache” and “dolor”.

The applications (designed for both patients and clinicians) focused on pain education, assessment and treatment were included.

**S3.C Review of the type of support of the apps available in stores**

A step-by-step sequential strategy was followed to assess the quality of the apps found in S3.B.

First, the name of each app was searched for in the following scientific databases (December 2013): Medline (National Library of Medicine), Science Citation Index Expanded (Web of Science), Health Reference Center Academic (Gale), Wiley Online Library, American Psychological Association (Psycinfo), SciVerse ScienceDirect (Elsevier), SpringerLink, Wolters Kluwer - Ovid - Lippincott Williams & Wilkins (CrossRef), Directory of Open Access Journals (DOAJ), Social Sciences Citation Index (Web of Science), Taylor & Francis Online - Journals, Expert Reviews (Future Science), Informa - Informa Healthcare (CrossRef), SpringerLink Open Access, Wolters Kluwer - Ovid (CrossRef), BMJ Journals, DiVA - Academic Archive Online, Informa (CrossRef).

Then, the web page “myhealthapps.net” was also reviewed. All the pain-related apps were recorded.

Finally, the name of each app was Google searched for such information as whether the developers had a webpage, which research centers used the app, who its creators were and/or the results it had provided, etc. This information was compared with the information obtained in S3.A to see if the authors of the apps were the same as the authors of the publications.

**S3.D Review of the scientifically assessed pain-related apps available in the stores**

Finally, the name of each app retrieved in S3.A (if provided) was searched for in each of the following shops (December 2013): App Store (iPhone), Blackberry App World, Google Play (Android), Nokia Store and Windows Phone Store.

This last step was conducted so that we did not miss any relevant app.
